# Neuronal pentraxin 2: a synapse-derived CSF biomarker in genetic frontotemporal dementia

**DOI:** 10.1136/jnnp-2019-322493

**Published:** 2020-04-09

**Authors:** Emma L van der Ende, Meifang Xiao, Desheng Xu, Jackie M Poos, Jessica L Panman, Lize C Jiskoot, Lieke H Meeter, Elise GP Dopper, Janne M Papma, Carolin Heller, Rhian Convery, Katrina Moore, Martina Bocchetta, Mollie Neason, Georgia Peakman, David M Cash, Charlotte E Teunissen, Caroline Graff, Matthis Synofzik, Fermin Moreno, Elizabeth Finger, Raquel Sánchez-Valle, Rik Vandenberghe, Robert Laforce Jr, Mario Masellis, Maria Carmela Tartaglia, James B Rowe, Christopher R Butler, Simon Ducharme, Alex Gerhard, Adrian Danek, Johannes Levin, Yolande AL Pijnenburg, Markus Otto, Barbara Borroni, Fabrizio Tagliavini, Alexandre de Mendonca, Isabel Santana, Daniela Galimberti, Harro Seelaar, Jonathan D Rohrer, Paul F Worley, John C van Swieten, Genetic Frontotemporal Dementia Initiative (GENFI), Martin N Rossor

**Affiliations:** 1 Department of Neurology and Alzheimer Center, Erasmus University Medical Center, Rotterdam, Netherlands; 2 Solomon H Snyder Department of Neuroscience, Johns Hopkins University School of Medicine, Baltimore, Maryland, United States; 3 Department of Radiology, Leiden University Medical Center, Leiden, Netherlands; 4 Dementia Research Centre, UCL Queen Square Institute of Neurology, London, United Kingdom; 5 Dementia Research Institute, Department of Neurodegenerative Disease, University College London, London, United Kingdom; 6 Neurochemistry Laboratory, Department of Clinical Chemistry, Amsterdam Neuroscience, Amsterdam University Medical Center, Amsterdam, Netherlands; 7 Karolinska Institutet, Dept NVS, Division of Neurogeriatrics, Bioclinicum, Stockholm, Sweden; 8 Unit of Hereditary Dementia, Theme Aging, Karolinska University Hospital-Solna, Stockholm, Sweden; 9 German Center for Neurodegenerative Diseases (DZNE), University of Tübingen, Tübingen, Germany; 10 Department of Neurodegenerative Diseases, Hertie Institute for Clinical Brain Research, Tübingen, Germany; 11 Department of Neurology, Donostia University Hospital, San Sebastian, Gipuzkoa, Spain; 12 Department of Clinical Neurological Sciences, University of Western Ontario, London, Ontario, Canada; 13 Alzheimer's Disease and Other Cognitive Disorders Unit, Hospital Clinic de Barcelona, Barcelona, Spain; 14 Laboratory for Cognitive Neurology, Department of Neurosciences, KU Leuven, Leuven, Belgium; 15 Clinique Interdisciplinaire de Mémoire du CHU de Québec, Département des Sciences Neurologiques, Université Laval, Québec, Quebec City, Canada; 16 Sunnybrook Health Sciences Centre, Sunnybrook Research Institute, University of Toronto, Toronto, Ontario, Canada; 17 Tanz Centre for Research in Neurodegenerative Disease, University of Toronto, Toronto, Ontario, Canada; 18 Cambridge University Centre for Frontotemporal Dementia, University of Cambridge, Cambridge, United Kingdom; 19 Department of Clinical Neurology, University of Oxford, Oxford, United Kingdom; 20 Montreal Neurological Institute and McGill University Health Centre, McGill University, Montreal, Québec, Canada; 21 Department of Nuclear Medicine and Geriatric Medicine, University Hospital Essen, Essen, Germany; 22 Divison of Neuroscience and Experimental Psychology, University of Manchester, Manchester, United Kingdom; 23 Neurologische Klinik und Poliklinik, Ludwig-Maximilians-Universität München, Munich, Germany; 24 German Center for Neurodegenerative Diseases, (DZNE), Munich, Germany; 25 Munich Cluster for Systems Neurology, (SyNergy), Munich, Germany; 26 Alzheimer Center Amsterdam, Department of Neurology, Amsterdam Neuroscience, Vrije Universiteit Amsterdam, Amsterdam UMC, Amsterdam, Netherlands; 27 Department of Neurology, Universität Ulm, Ulm, Germany; 28 Centre for Neurodegenerative Disorders, Neurology Unit, Department of Clinical and Experimental Sciences, University of Brescia, Brescia, Italy; 29 Fondazione IRCCS, Istituto Neurologico Carlo Besta, Milan, Italy; 30 Faculty of Medicine, University of Lisbon, Lisbon, Portugal; 31 Center for Neuroscience and Cell Biology, Faculty of Medicine, University of Coimbra, Coimbra, Portugal; 32 Department of Neurological Sciences, Dino Ferrari Center, University of Milan, Milan, Italy; 33 Fondazione IRCCS Ca' Granda, Ospedale Maggiore Policlinico, Milan, Italy; 34 Department of Neurology, Johns Hopkins University School of Medicine, Baltimore, Maryland, United States

## Abstract

**Introduction:**

Synapse dysfunction is emerging as an early pathological event in frontotemporal dementia (FTD), however biomarkers are lacking. We aimed to investigate the value of cerebrospinal fluid (CSF) neuronal pentraxins (NPTXs), a family of proteins involved in homeostatic synapse plasticity, as novel biomarkers in genetic FTD.

**Methods:**

We included 106 presymptomatic and 54 symptomatic carriers of a pathogenic mutation in *GRN*, *C9orf72* or *MAPT*, and 70 healthy non-carriers participating in the Genetic Frontotemporal dementia Initiative (GENFI), all of whom had at least one CSF sample. We measured CSF concentrations of NPTX2 using an in-house ELISA, and NPTX1 and NPTX receptor (NPTXR) by Western blot. We correlated NPTX2 with corresponding clinical and neuroimaging datasets as well as with CSF neurofilament light chain (NfL) using linear regression analyses.

**Results:**

Symptomatic mutation carriers had lower NPTX2 concentrations (median 643 pg/mL, IQR (301–872)) than presymptomatic carriers (1003 pg/mL (624–1358), p<0.001) and non-carriers (990 pg/mL (597–1373), p<0.001) (corrected for age). Similar results were found for NPTX1 and NPTXR. Among mutation carriers, NPTX2 concentration correlated with several clinical disease severity measures, NfL and grey matter volume of the frontal, temporal and parietal lobes, insula and whole brain. NPTX2 predicted subsequent decline in phonemic verbal fluency and Clinical Dementia Rating scale plus FTD modules. In longitudinal CSF samples, available in 13 subjects, NPTX2 decreased around symptom onset and in the symptomatic stage.

**Discussion:**

We conclude that NPTX2 is a promising synapse-derived disease progression biomarker in genetic FTD.

## Introduction

Frontotemporal dementia (FTD), a common form of early-onset dementia, has an autosomal dominant inheritance in 20%–30% of patients, most often due to mutations in granulin (*GRN*), chromosome 9 open reading frame 72 (*C9orf72*) or microtubule-associated protein tau (*MAPT*).^1^ Developing sensitive biomarkers to detect disease onset at an early, even preclinical stage is of utmost importance for upcoming therapeutic interventions. Genetic forms of FTD provide a unique opportunity to study disease progression from presymptomatic to overt FTD and to identify novel biomarkers.

Our previous proteomics study identified neuronal pentraxin receptor (NPTXR) in cerebrospinal fluid (CSF) as the most promising candidate biomarker in genetic FTD, with markedly reduced levels in the symptomatic stage.^2^ NPTXR forms complexes with NPTX1 and NPTX2 (also termed neuronal activity related protein, Narp) at excitatory synapses of pyramidal neurons onto parvalbumin interneurons and contributes to synaptic homeostatic plasticity.^3 4^ Increasing evidence suggests that dysfunction and degeneration of synapses is an early pathological event in FTD,^5–7^ especially in *GRN*-associated FTD,^8–10^ a concept widely recognised in other neurodegenerative diseases.^5 11^ Fluid biomarkers reflecting synaptic integrity in FTD might therefore contribute to an early diagnosis and monitoring of disease progression in clinical practice and clinical trials. Following studies in Alzheimer’s disease (AD) that identified NPTXs as candidate biomarkers,^12–20^ we hypothesised that NPTXs could be valuable synapse-derived biomarkers in genetic FTD.

In the present study, we measured CSF NPTXs in a large cohort of *GRN*, *C9orf72* and *MAPT* mutation carriers participating in the international Genetic FTD Initiative (GENFI). We focused our attention on NPTX2, as the available ELISA for NPTX2 allowed for more accurate quantitative measurements than the Western blots used for NPTX1 and NPTXR. We explored the relationship between NPTX2 and clinical disease severity, grey matter volume and CSF neurofilament light chain (NfL), a marker of neuroaxonal damage.^21^

## Methods

### Subjects

Subjects were included from 16 centres across Europe and Canada participating in GENFI, a longitudinal cohort study of patients with FTD due to a pathogenic mutation in *GRN*, *C9orf72* or *MAPT* and healthy 50% at-risk relatives (either presymptomatic mutation carriers or non-carriers). Participants underwent an annual assessment as previously described,^22^ including neurological and neuropsychological examination, MRI of the brain, and collection of blood and CSF. Knowledgeable informants completed questionnaires about potential changes in cognition or behaviour.

For the present study, we included all participants with at least one CSF sample, amounting to 54 symptomatic mutation carriers (15 *GRN*, 31 *C9orf72*, 8 *MAPT*), 106 presymptomatic mutation carriers (47 *GRN*, 42 *C9orf72*, 17 *MAPT*) and 70 non-carriers. Longitudinal CSF samples were available in 13 subjects.

Mutation carriers were considered symptomatic if they fulfilled international consensus criteria for FTD.^23 24^ Furthermore, as *C9orf72* mutations are also associated with amyotrophic lateral sclerosis (ALS), which is increasingly considered part of the FTD disease spectrum,^1^ *C9orf72* mutation carriers fulfilling criteria for ALS,^25^ but not FTD, were also considered symptomatic. We calculated disease duration based on a caregiver’s estimation of the emergence of first symptoms.

Global cognition was scored using the Mini Mental State Examination (MMSE) and Clinical Dementia Rating scale (CDR) plus FTD modules.^26^ The Revised Cambridge Behavioural Inventory (CBI-R) was used to measure behavioural changes.^27^ The Trail Making Test part B (TMT-B) and phonemic verbal fluency were included as measures of executive functioning.^28^ TMT-B was truncated to 300 s for subjects that exceeded the time limit. All scores were collected within 6 months of CSF collection.

T1-weighted MRI on three Tesla scanners was obtained within 6 months of CSF collection in 190 participants (35 symptomatic and 91 presymptomatic mutation carriers, 64 non-carriers). All MRI scans were acquired using a standardised GENFI protocol.^22^ T1-weighted volumetric MRI scans were parcellated into brain regions as previously described,^22^ using an atlas propagation and fusion strategy^29^ to generate volumes of the whole brain, frontal, temporal, parietal and occipital lobes, insula and cingulate gyrus. Brain volumes were expressed as a percentage of total intracranial volume (TIV), computed with SPM12 running under Matlab R2014b (Math Works, Natick, Massachusetts, USA).^30^

### Sample collection and laboratory methods

CSF was collected in polypropylene tubes, centrifuged and stored at −80°C within 2 hours of withdrawal according to a standardised GENFI protocol.

NPTX2 concentrations were measured using an in-house ELISA as described previously.^12^ The intra-assay and interassay coefficients of variation (CV) were <2% and<5%, respectively. The lower limit of quantification (LLOQ) was 5 pg/mL; all NPTX2 measurements were above the LLOQ. NPTX1 and NPTXR were measured by Western blot. Rabbit anti-NPTX1 was described previously^3^; sheep anti-NPTXR antibody is from R&D systems (Cat. Number: AF4414; RRID: AB_2153869). Immunoreactive bands were visualised by the enhanced chemiluminescent substrate (ECL, Pierce) on X-ray film and quantified using the image software TINA (www.tina-vision.net). Western blot results were expressed as a percentage of abundancy compared with non-carriers, that is, mean abundancy in non-carriers was set at 100%. Detailed methods are reported in online supplementary file 1.

10.1136/jnnp-2019-322493.supp1 Supplementary data

All NPTX measurements were performed in two batches in the Neuroscience Laboratory at Johns Hopkins University, Baltimore, USA. The mean CV of NPTX2 of the two batches was 5.4%. Longitudinal measurements were performed in one batch.

CSF NfL concentrations were measured in duplicate in one batch using the Simoa NF-Light Advantage Kit from Quanterix on a Simoa HD-1 analyser instrument according to manufacturer’s instructions. The mean CV of duplicate measurements was 3.2% (range 0.1%–15.6%). NfL measurements were missing in four subjects (two symptomatic *GRN* and two symptomatic *C9orf72* mutation carriers) due to insufficient CSF.

### Standard protocol approvals and patient consents

Clinical researchers were blinded to the genetic status of at-risk individuals unless they had undergone predictive testing. Laboratory technicians were blinded to all clinical and genetic information.

### Statistical analysis

Statistical analyses were performed in IBM SPSS Statistics V.24 and *R*. Graphs were drafted in *R* and GraphPad Prism 8. Statistical significance was set at 0.05 (two-sided). The primary analysis in the study was to investigate whether NPTX2, NPTX1 and NPTXR concentration differ among symptomatic mutation carriers, presymptomatic mutation carriers and non-carriers. We restricted correlative analyses of clinical and neuroimaging parameters to NPTX2 because an ELISA was available, which is more sensitive for quantitative analyses than Western blots used for NPTX1 and NPTXR.

Demographic and clinical variables were compared between groups using Kruskal-Wallis tests for continuous variables and a χ^2^ test for sex. Normality of biomarker data was assessed using Kolmogorov-Smirnov tests and visual inspection of Q-Q plots. While raw biomarker values were not normally distributed, normal distributions were achieved after square-root transformation of NPTX2 and log-transformation of NfL. We performed analyses of covariance (ANCOVAs) on transformed biomarker values with age as a covariate to test for group differences. In comparisons between symptomatic mutation carriers, we also included disease duration as a covariate.

The diagnostic performance of NPTX2 to discriminate between the three clinical groups (symptomatic mutation carriers, presymptomatic mutation carriers, non-carriers) was assessed by the area under the curve (AUC) of receiver operating characteristic analyses, with optimal cut-off levels determined by the highest Youden’s index.^31^

Linear regression models were constructed to study the relationship between NPTX2 concentration (dependent variable; square-root transformed to meet model assumptions) and (1) regional grey matter volume, (2) clinical disease severity measures and (3) NfL, with age, gender and study site as covariates. For analyses of cognitive tests (MMSE, TMT-B, phonemic verbal fluency) we also included years of education as a covariate. All analyses were performed for mutation carriers combined and for symptomatic and presymptomatic mutation carriers separately. Correction for multiple comparisons was done with the Bonferroni method.

Additional linear regression models were constructed to test whether NPTX2 could predict subsequent cognitive decline, as measured by annualised changes in clinical disease severity scores (score at the time of CSF collection subtracted from a later score and divided by time interval), correcting for age, gender and study site and in cognitive tests for years of education.

Due to the limited sample size, statistical analyses on longitudinal NPTX2 measurements were limited to exploratory correlations between changes in NPTX2 and time interval, to test for an overall trend in NPTX2 concentration over time.

### Data availability

The raw data of this project is part of GENFI and de-identified patient data can be accessed on reasonable request (j.c.vanswieten@erasmusmc.nl and j.rohrer@ucl.ac.uk).

## Results

### Demographic and clinical data

Subject characteristics are shown in table 1. Symptomatic mutation carriers were significantly older than presymptomatic carriers and non-carriers, both overall and for each genetic group (*GRN, C9orf72* and *MAPT*) separately. Three presymptomatic mutation carriers converted to the symptomatic stage (‘converters’) during follow-up (2 *GRN*, 1 *MAPT*).

**Table 1:** Subject characteristics. The clinical phenotype of symptomatic mutation carriers was behavioural variant FTD (n=37), primary progressive aphasia (n=7), FTD with amyotrophic lateral sclerosis (ALS) (n=4), ALS without FTD (n=3), memory-predominant FTD (n=1), progressive supranuclear palsy (n=1) and dementia not otherwise specified (n=1). Continuous variables are reported as medians±IQR

	Non-carriers	Presymptomatic carriers	Symptomatic carriers	P value
N	70	106	54	–
Sex, male (%)	31 (44%)	47 (44%)	32 (59%)	0.157*
Age at CSF collection, years	47 (40–58)	45 (34–56)	63 (56–69)	<0.001†
MMSE	30 (29–30)	30 (29–30)	26 (24–28)	<0.001†
CDR plus FTD modules	0 (0–0)	0 (0–0)	9 (3–10)	<0.001†
Disease duration, years	–	–	3 (2–6)	–
NPTX2, pg/mL	990 (597–1373)	1003 (624–1358)	643 (301–872)	<0.001‡

Gene-specific information	*GRN*	*C9orf72*	*MAPT*	*GRN*	*C9orf72*	*MAPT*	P value
N	47	42	17	15	31	8	–
Age at CSF collection, years	54 (41–58)	43 (32–53)	42 (34–46)	64 (61–69)	60 (55–70)	61 (53–64)	<0.001†
NPTX2, pg/mL	1072 (661–1406)	901 (534–1387)	1079 (389–1263)	741 (385–870)	609 (305–884)	561 (233–861)	<0.001‡
Age at symptom onset, years	–	–	–	63 (54–66)	55 (49–62)	55 (52–58)	0.109†
Disease duration, years	–	–	–	2 (1–4)	4 (2–8)	3 (1–8)	0.238†

*χ^2^ test.

†Kruskall-Wallis tests.

‡ANCOVA with age as covariate.

ANCOVA, Analysis of covariance; CDR, Clinical Dementia Rating scale; CSF, cerebrospinal fluid; FTD, frontotemporal dementia; MMSE, Mini Mental State Examination; NPTX, neuronal pentraxin.

### NPTX2 concentration

**Figure 1:**
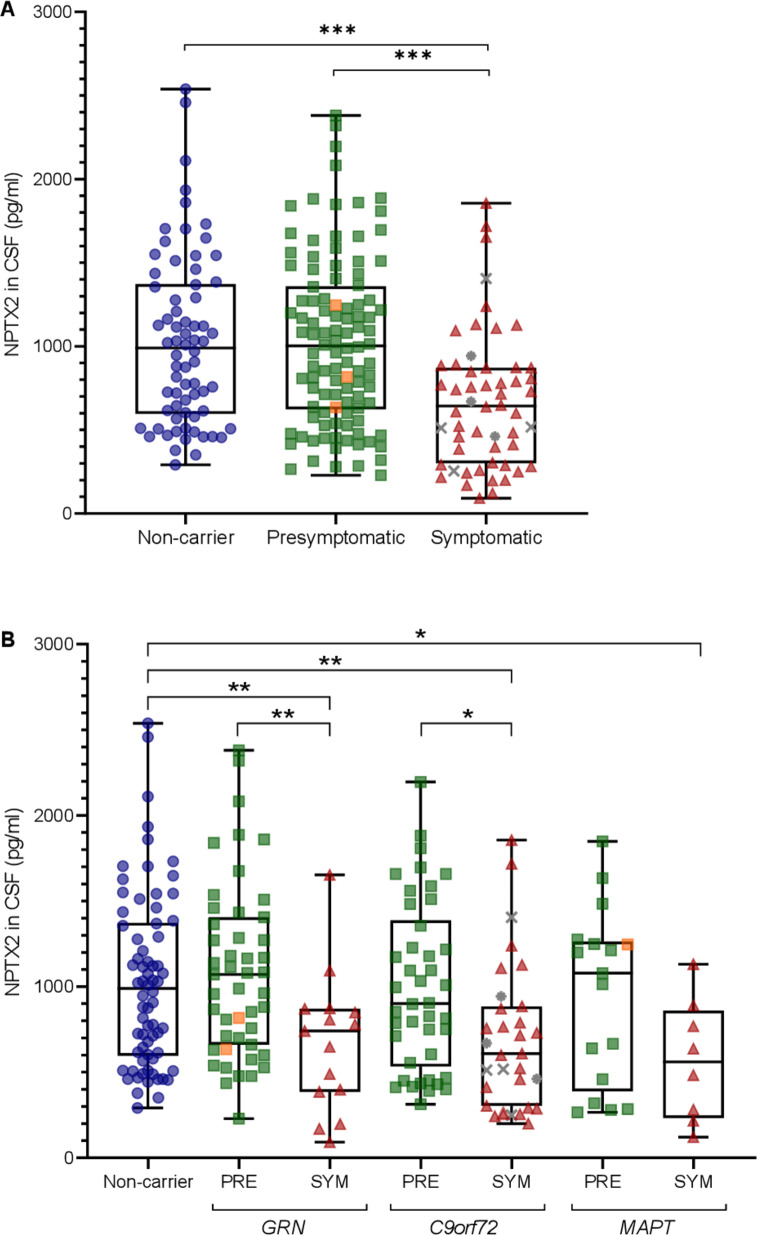
NPTX2 levels (A) in presymptomatic (n=106) and symptomatic mutation carriers (n=54) and non-carriers (n=70) and (B) separated by genetic group. Whiskers indicate minimum and maximum values. Orange squares indicate subjects who converted to the symptomatic stage during follow-up (n=3); grey asterisks indicate subjects with amyotrophic lateral sclerosis (ALS) without frontotemporal dementia (FTD) (n=3); grey crosses indicate subjects with both FTD and ALS (n=4). P values are from analyses of covariance (ANCOVAs) with age as a covariate and Bonferroni correction for multiple comparisons. *p<0.05; **p<0.01; ***p<0.001. CSF, cerebrospinal fluid; NPTX, neuronal pentraxin; PRE, presymptomatic mutation carrier; SYM: symptomatic mutation carrier.

Overall, NPTX2 levels were lower in symptomatic mutation carriers (median 643 pg/mL, IQR 301–872) than in presymptomatic carriers (1003 pg/mL (624–1358); p<0.001) and non-carriers (990 pg/mL (597–1373); p<0.001) (figure 1A). NPTX2 levels did not differ significantly between presymptomatic mutation carriers and non-carriers (p=0.859).

When analysed per genetic group, symptomatic *GRN* and *C9orf72* mutation carriers had significantly lower NPTX2 levels than their presymptomatic counterparts (*GRN*: 741 pg/mL vs 1072 pg/mL; p=0.003; *C9orf72*: 609 pg/mL vs 901 pg/mL; p=0.023) and non-carriers (*GRN*: p=0.007; *C9orf72*: p=0.004) (table 1, figure 1B). Symptomatic *MAPT* mutation carriers had lower NPTX2 levels than non-carriers (561 pg/mL; p=0.027) but not compared with presymptomatic *MAPT* mutation carriers (1079 pg/mL; p=0.213). NPTX2 levels did not differ between symptomatic carriers of different genetic groups (p=0.709). Similar results were obtained after excluding mutation carriers with ALS without FTD (n=3).

Overall, a correlation was found between NPTX2 and age (r_s_=−0.141; p=0.033) (online supplementary figure 1); this correlation was also found in mutation carriers alone (r_s_=−0.205; p=0.009) but not in non-carriers alone (r_s_=0.070; p=0.566). NPTX2 levels did not differ by sex (p=0.976).

10.1136/jnnp-2019-322493.supp2 Supplementary data

### Diagnostic accuracy of NPTX2

The AUC for NPTX2 to distinguish symptomatic from presymptomatic mutation carriers was 0.71 (95% CI 0.63 to 0.80), with an optimal cut-off of 895 pg/mL (sensitivity 82%, specificity 56%) (online supplementary figure 2). The AUC to distinguish symptomatic mutation carriers from non-carriers was 0.71 (95% CI 0.61 to 0.80), with an optimal cut-off of 945 pg/mL (sensitivity 83%, specificity 53%). NPTX2 did not distinguish presymptomatic mutation carriers from non-carriers (AUC 0.50 (95% CI 0.41 to 0.58)).

10.1136/jnnp-2019-322493.supp3 Supplementary data

### NPTX2 and clinical and neuroimaging data

**Figure 2:**
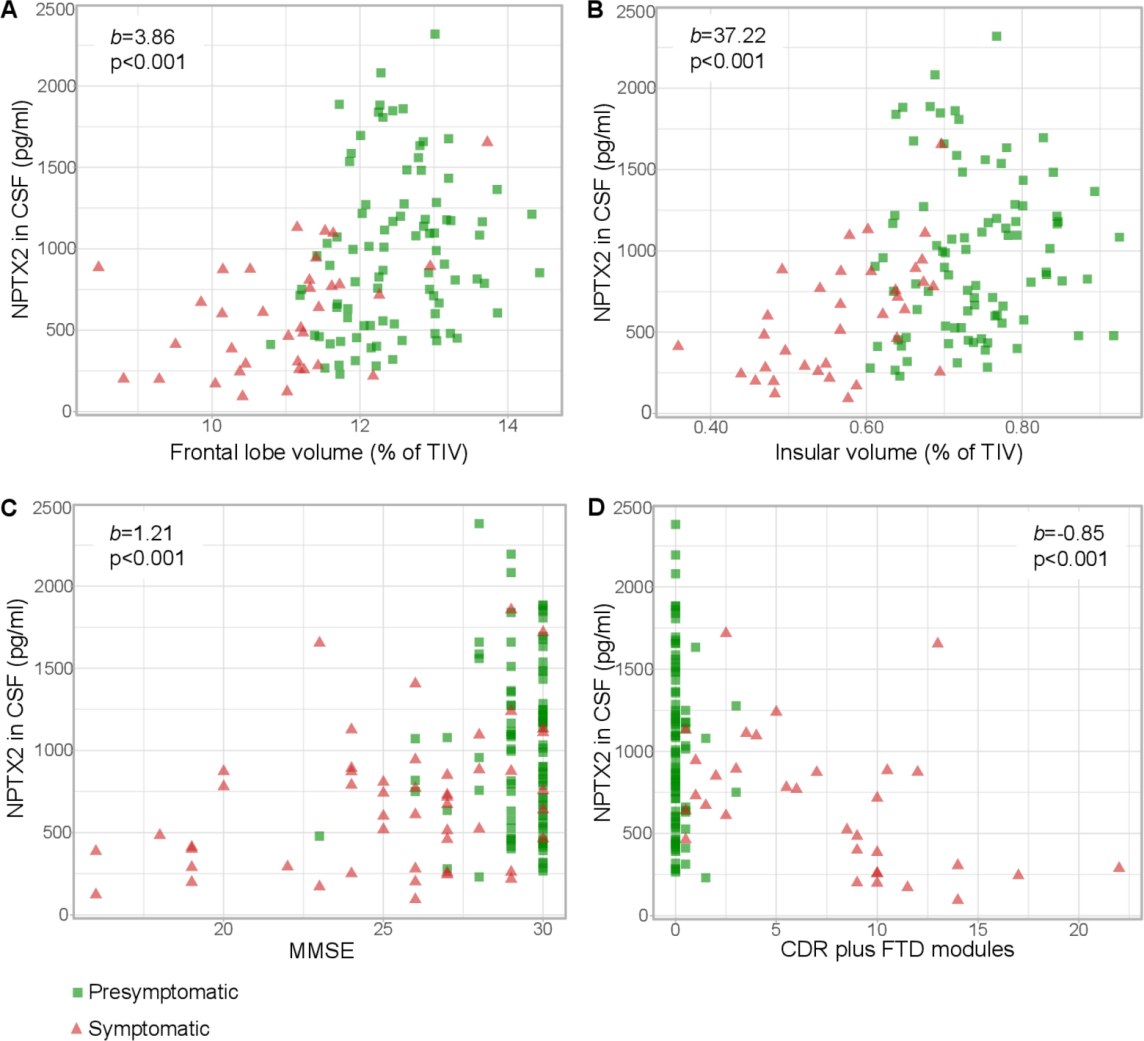
Relationship between NPTX2 and (A) frontal lobe volume (n=126), (B) insular volume (n=126), (C) Mini Mental State Examination (MMSE) (n=145) and (D) Clinical Dementia Rating scale (CDR) plus FTD modules (n=120) among mutation carriers. b and p were obtained through multiple linear regression with square-root transformed NPTX2 as the dependent variable, adjusting for age, gender and study site; for MMSE, we also included years of education as a covariate. CSF, cerebrospinal fluid; FTD, frontotemporal dementia; NPTX, neuronal pentraxin; TIV, total intracranial volume.

NPTX2 levels in mutation carriers correlated significantly with grey matter volume of the whole brain, frontal, temporal and parietal lobes and insula, and in symptomatic mutation carriers alone, with whole brain volume, frontal lobe and insular volume (figure 2A, B, table 2, online supplementary figure 3). In presymptomatic mutation carriers, NPTX2 levels were associated with frontal lobe volume but this was no longer significant after multiple testing correction (table 2). Among non-carriers (n=64), no significant associations were found with any of the regional grey matter volumes (online supplementary table 1). Results were unchanged after repeating the analyses with raw grey matter volumes (ie, not corrected for TIV).

10.1136/jnnp-2019-322493.supp4 Supplementary data

**Table 2:** Relationship between NPTX2 and grey matter volume. Results were obtained through multiple linear regression with square-root transformed NPTX2 as dependent variable, adjusting for age, gender and study site. All grey matter volumes were corrected for total intracranial volume

	All mutation carriers (n=126)	Symptomatic mutation carriers (n=35)	Presymptomatic mutation carriers (n=91)
Whole brain volume			
b (SE)	1.07 (0.25)	1.21 (0.42)	0.58 (0.39)
β	0.533	0.585	0.215
p	<0.001*	0.007*	0.145
Frontal lobe			
b (SE)	3.86 (0.83)	4.40 (1.33)	3.48 (1.44)
β	0.516	0.623	0.331
p	<0.001*	0.003*	0.018
Temporal lobe			
b (SE)	4.01 (1.32)	5.00 (2.27)	0.67 (2.11)
β	0.338	0.448	0.039
p	0.003*	0.037	0.752
Parietal lobe			
b (SE)	4.89 (1.77)	5.51 (4.25)	2.70 (2.18)
β	0.323	0.319	0.169
p	0.007*	0.208	0.218
Occipital lobe			
b (SE)	1. 45 (1.77)	-6.65 (4.03)	3.10 (1.97)
β	0.086	-0.341	0.185
p	0.412	0.112	0.119
Cingulate gyrus			
b (SE)	10.49 (5.27)	14.72 (11.32)	0.46 (7.27)
β	0.205	0.285	0.008
p	0.049	0.206	0.95
Insula			
b (SE)	37.22 (8.69)	69.21 (13.09)	12.61 (13.16)
β	0.482	0.786	0.119
p	<0.001*	<0.001*	0.341

P values marked with an asterisk remained significant after Bonferroni correction. b indicates unstandardised regression coefficient. β indicates standardised regression coefficient.

NTPX, neuronal pentraxin.

Among mutation carriers, NPTX2 levels correlated with MMSE, TMT-B, phonemic verbal fluency, CDR plus FTD modules and CBI-R (table 3, figure 2C, D). In symptomatic mutation carriers alone, these correlations were similarly present for MMSE, TMT-B, CDR plus FTD modules and CBI-R. In presymptomatic mutation carriers alone, an association was found for TMT-B, which was no longer statistically significant after correction for multiple testing (table 3).

**Table 3:** Relationship between NPTX2 and disease severity scores, obtained through multiple linear regression with square-root transformed NPTX2 as dependent variable, adjusting for age, gender, study site and, in analyses of MMSE, TMT-B and letter fluency, years of education

	All mutation carriers	Symptomatic mutation carriers	Presymptomatic mutation carriers
MMSE			
n	145	50	95
b (SE)	1.21 (0.26)	0.98 (0.29)	1.58 (0.79)
β	0.442	0.467	0.23
p	<0.001*	0.002*	0.051
TMT-B			
n	125	34	91
b (SE)	−0.04 (0.01)	−0.02 (0.02)	−0.09 (0.04)
β	−0.353	−0.234	−0.259
p	<0.001*	<0.001*	0.03
Phonemic verbal fluency			
n	132	39	93
b (SE)	0.14 (0.05)	0.15 (0.10)	0.05 (0.08)
β	0.255	0.25	0.076
p	0.009*	0.151	0.536
CDR plus FTD modules			
n	120	33	87
b (SE)	−0.85 (0.19)	−0.72 (0.24)	−1.19 (1.67)
β	−0.435	−0.479	−0.078
p	<0.001*	0.007*	0.478
CBI-R			
n	119	40	79
b (SE)	−0.13 (0.03)	−0.10 (0.04)	−0.02 (0.14)
β	−0.489	−0.394	−0.014
p	<0.001*	0.017*	0.906

P values are before multiple testing correction; p values marked with an asterisk remained significant after Bonferroni correction. b indicates unstandardised regression coefficient; β indicates standardised regression coefficient.

CBI-R, Revised Cambridge Behavioural Inventory; CDR, Clinical Dementia Rating scale; MMSE, Mini Mental State Examination; TMT-B, Trail Making Test part B.

### NPTX2 and NFL concentration

Symptomatic mutation carriers had significantly higher NfL levels than presymptomatic carriers (2575 pg/mL (1218–4592) vs 471 pg/mL (305–729); p<0.001) and non-carriers (421 pg/mL (298–555); p<0.001). These differences were also found for each genetic group separately (online supplementary figure 4). No differences were seen between presymptomatic mutation carriers and non-carriers (p=0.517). Similar results were obtained after exclusion of nine outliers (values>3*IQR from the median) and after exclusion of subjects with ALS without FTD (n=3).

10.1136/jnnp-2019-322493.supp5 Supplementary data

**Figure 3:**
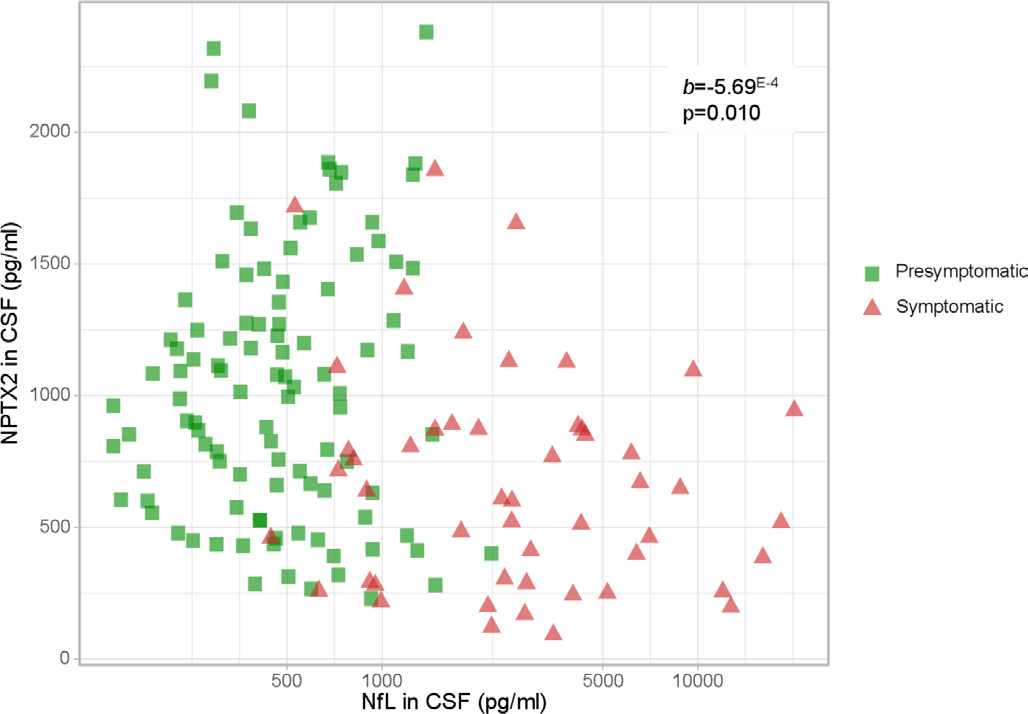
Correlation between NPTX2 and NfL levels in presymptomatic (n=106) and symptomatic (n=50) mutation carriers. NfL is plotted on a log-scale for visualisation purposes. b and p were obtained through multiple linear regression with square-root transformed NPTX2 as the dependent variable, adjusting for age, gender and study site. CSF, cerebrospinal fluid; NfL, neurofilament light chain; NPTX, neuronal pentraxin.

NPTX2 concentration across all mutation carriers was inversely associated with NfL concentration (b=−5.69E-4; p=0.010; n=156) (figure 3). This correlation was not observed for symptomatic (b*=*−3.61E-4; p=0.141; n=50) or presymptomatic mutation carriers (b=−3.64E-5; p=0.971; n=106) alone. Repeating the analyses for symptomatic mutation carriers after exclusion of patients with concomitant or isolated ALS revealed a trend towards association between NfL and NPTX2 (b=−7.55E-4; p=0.061; n=43).

### Longitudinal NPTX2 measurements

Longitudinal CSF samples were available in 10 presymptomatic mutation carriers (of whom two had converted to the symptomatic stage at follow-up CSF collection), two symptomatic mutation carriers and one non-carrier. The median time between samples was 2.0 years (IQR 1.8–2.1).

**Figure 4:**
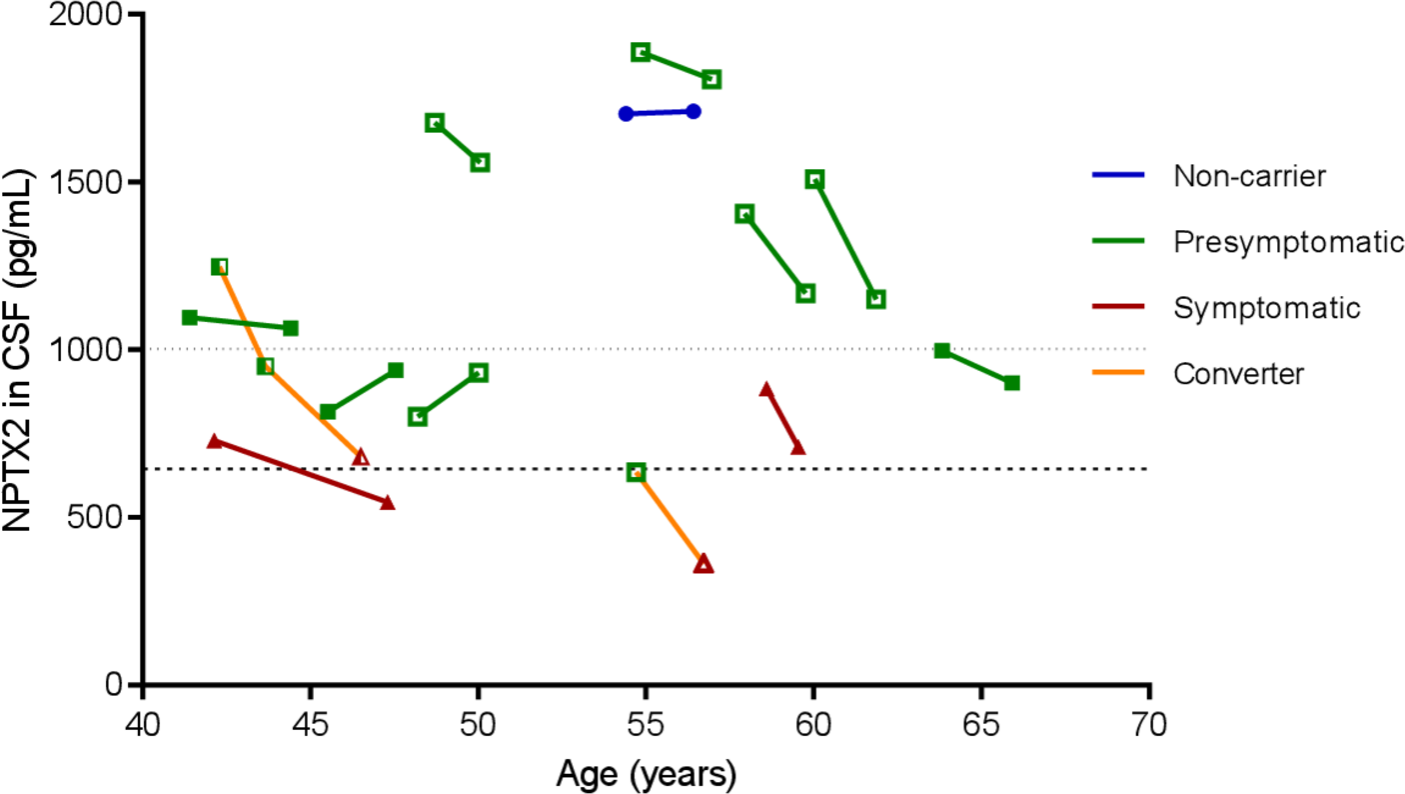
Longitudinal NPTX2 levels plotted against age in 13 subjects with multiple CSF samples. A line is drawn between NPTX2 levels of follow-up samples. Presymptomatic samples are shown as green squares, symptomatic samples as red triangles and the non-carrier as blue circles. *GRN* mutation carriers are shown as open symbols, *C9orf72* mutation carriers as filled symbols and the *MAPT* mutation carrier as half-filled symbols. Dotted horizontal line indicates median NPTX2 level in presymptomatic mutation carriers (1003 pg/mL); dashed horizontal line indicates median in symptomatic mutation carriers (644 pg/mL). For blinding purposes, a jitter of ±2 years was applied to all subjects. CSF, cerebrospinal fluid; NPTX, neuronal pentraxin.

In the *MAPT* converter, visually, a decrease in NPTX2 was observed in two presymptomatic samples, with a further decrease in the symptomatic sample (figure 4). In the *GRN* converter, NPTX2 was already below the proposed cut-off level 1.2 years before symptom onset, with a further decrease in the symptomatic sample. Both symptomatic mutation carriers demonstrated NPTX2 decreases over time. Lower NPTX2 levels at follow-up were observed in all presymptomatic mutation carriers over the age of 50 years, while in younger presymptomatic carriers, NPTX2 trajectories seemed to be more varied. NPTX2 in one non-carrier subject was visually stable (figure 4).

There was no correlation between change in NPTX2 levels and time interval between CSF collections (r_s_=−0.116; p=0.705) (online supplementary figure 5).

10.1136/jnnp-2019-322493.supp6 Supplementary data

### NPTX2 and subsequent cognitive decline

NPTX2 was significantly associated with annualised change in phonemic verbal fluency (b=0.004; p=0.001; n=118), and CDR plus FTD modules (b=−0.001; p=0.025; n=105). The former remained significant after adjusting for clinical status, frontal lobe volume and CSF NfL. A trend was found for annualised change in MMSE (b=0.001; p=0.077; n=136). NPTX2 level was not associated with annualised change in CBI-R (p=0.200; n=93) or TMT-B (p=0.693; n=107) (online supplementary table 2, figure 6).

10.1136/jnnp-2019-322493.supp7 Supplementary data

### NPTX1 and NPTXR concentration

**Figure 5:**
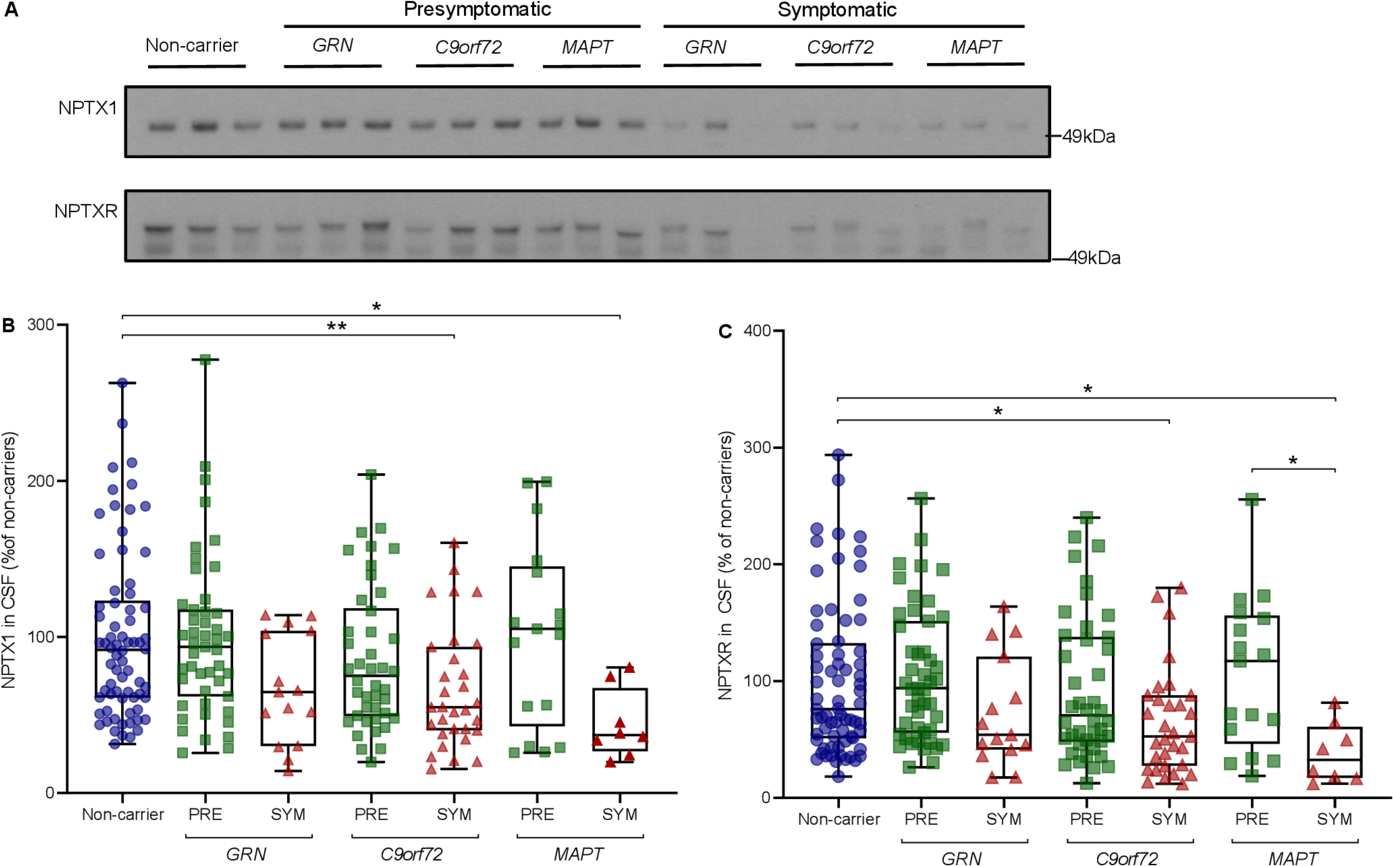
NPTX1 and NPTXR levels as measured by Western blot. (A) Representative (cropped) Western blots of NPTX1 and NPTXR (n=230); (B, C) NPTX1 and NPTXR levels across groups, expressed as a percentage of band intensity compared with non-carriers. Whiskers indicate minimum and maximum values. Displayed significance levels are from analysis of covariance (ANCOVA) on square-root transformed relative band intensities of NPTX1 and NPTXR with age as a covariate. *p<0.05; **p<0.01. CSF, cerebrospinal fluid; NPTX, neuronal pentraxin; PRE, presymptomatic mutation carrier; SYM, symptomatic mutation carrier.

NPTX1 levels were significantly lower in symptomatic mutation carriers compared with presymptomatic carriers (median 54% (36–88) vs 86% (55–118); p<0.001) and non-carriers (92% (61–123); p<0.001). Similar results were found for NPTXR (symptomatic vs presymptomatic: 51% (27–85) vs 81% (51–147); p=0.002; symptomatic vs non-carriers: 51% vs 76% (51–133); p<0.001) (figure 5). Separated by genetic group, symptomatic *C9orf72* and *MAPT* mutation carriers had significantly lower NPTX1 and NPTXR levels than non-carriers. In *GRN* mutation carriers, a similar pattern was observed, although not statistically significant. NPTX1 and NPTXR levels were strongly correlated with NPTX2 (r_s_=0.828 and r_s_=0.850, respectively, both p<0.001) (online supplementary figure 7).

10.1136/jnnp-2019-322493.supp8 Supplementary data

## Discussion

The present study of a large international cohort of genetic FTD reports low levels of NPTX2, NPTX1 and NPTXR in the symptomatic stage, with correlations between NPTX2 and both disease severity and grey matter volume. We propose NPTX2 as a novel synapse-derived biomarker of disease progression in genetic FTD.

The decreased levels of CSF NPTXs in patients with *GRN*-associated, *C9orf72*-associated and *MAPT*-associated FTD probably reflect a loss or dysfunction of their synaptic sources.^32^ Complexes of NPTXs accumulate at excitatory synapses between glutamatergic pyramidal neurons and parvalbumin-expressing (PV) interneurons in the cerebral cortex, hippocampus and cerebellum.^32^ NPTXs modulate the strength of these synapses through recruitment of AMPA-type glutamate receptors (AMPARs) to the postsynaptic membrane, thereby regulating excitatory drive of pyramidal neurons onto PV-interneurons.^4 12 32^ PV-interneurons in turn contact surrounding pyramidal neurons and prevent neural circuits from becoming too active (diagram provided in Hanson^33^).^34^ The expression of NPTX2—but not NPTX1 or NPTXR—is induced by synapse activity,^3 35^ and the relative ratio of the NPTXs in the complex is dynamically dependent on the neuron’s activity. Of the three proteins, NPTX2 is most effective at AMPAR recruitment, but their combined expression is synergistic.^3^ *NPTX2* knockout mice have reduced AMPARs, resulting in less inhibitory PV-interneuron activity; the subsequent disruption of pyramidal neuron—PV-interneuron circuits is thought to underlie cognitive impairment, and NPTX2 loss has been hypothesised to drive neurodegeneration.^12^ Restoring these circuits is emerging as a potential treatment strategy in neurodegenerative disease^36^; in this regard, measuring NPTX2 in CSF could be especially relevant to select and monitor patients with aberrant pyramidal neuron-PV interneuron circuits.^12 36^ The lack of correlation in the current study between NPTX2 and brain volume in healthy non-carriers reflects that NPTX2 is not merely a measure of synapse density.

The present study reports low NPTX2 levels in all included forms of genetic FTD. The lack of significant differences between presymptomatic and symptomatic *MAPT* mutation carriers likely reflects insufficient statistical power given the small sample size, or could reflect differences in underlying pathology (eg, tau pathology in *MAPT-*associated FTD vs TDP-43 pathology in *GRN*-associated and *C9orf72-*associated FTD).^1^ While these findings are novel in FTD, a few studies have identified reduced NPTXs in AD, both in brain and in CSF,^12–19^ although mostly through mass spectrometry approaches (box 1). The observed reductions in both FTD and AD suggest that reduced NPTXs reflect general rather than gene-specific or disease-specific pathological alterations. To date, CSF NPTX2 levels in other neurodegenerative diseases have not been reported; future research should focus on the measurement of CSF NPTXs across a broader range of neurodegenerative diseases, including sporadic FTD and Parkinson’s disease.^37^

Box 1 **CSF NPTXs in other neurodegenerative diseases**

- Cerebrospinal fluid (CSF) neuronal pentraxin (NPTX)2 levels are reduced in patients with Alzheimer’s disease (AD) compared with controls.^12 14 17^ In AD, low CSF NPTX2 levels are associated with cognitive impairment and subsequent memory decline, as well as hippocampal atrophy and subsequent medial temporal lobe atrophy.^12 17^
- To date, CSF NPTX2 levels have not been reported in other neurodegenerative diseases, such as sporadic FTD and Parkinson’s disease.
- Differentially regulated levels of CSF NPTX1 and NPTXR have been reported in patients with AD, mostly identified through mass spectrometry approaches.^13 15 16 18–20^ In presymptomatic stages of autosomal dominant AD, mild cognitive impairment and early-stage AD, a transient increase in NPTXs has been observed.^15 16 19 20^
- In brain tissue, NPTX2 levels are decreased in patients with AD.^12^ Conversely, one study has reported accumulation of NPTX2 in Lewy bodies in patients with Parkinson’s disease.^37^

The correlations between NPTX2 concentration and several disease severity measures suggest that NPTX2 might further decrease with disease progression. This is supported by longitudinal NPTX2 decreases over time in two symptomatic mutation carriers and could reflect a link between progressive synapse pathology and cognitive decline; more longitudinal data is needed to confirm this. The association between NPTX2 levels and subsequent decline in phonemic verbal fluency and CDR plus FTD modules indicates that NPTX2 may have prognostic significance and is in line with previous findings in AD. The correlations between NPTX2 and grey matter volume in regions typically affected in FTD, including the frontal lobe and insula,^23^ are comparable to the previously reported correlations with hippocampal volume in AD and provide further evidence for NPTX2 as a disease progression marker.^12 17^

Longitudinally, we observed strong NPTX2 decreases in two converters; in the *MAPT* converter, this decrease was already observed in two presymptomatic samples; similarly, in the *GRN* converter, NPTX2 levels were already low in the presymptomatic sample. Although these results must be interpreted with caution due to the small sample size, they provide tentative evidence that NPTX2 might be an early disease marker. The overall lack of differences in NPTXs between presymptomatic mutation carriers and non-carriers might reflect the inclusion of mutation carriers of all ages; therefore, time to symptom onset was highly variable. Remarkably, in presymptomatic autosomal dominant AD, mild cognitive impairment and early stage AD, a transient increase in NPTXs has been observed, with a subsequent decline as the disease progresses.^15 16 19 20^ This discrepancy in NPTXs dynamics may result from differences in underlying pathophysiology.

The diagnostic accuracy of NPTX2 of 71% to distinguish symptomatic from presymptomatic mutation carriers is comparable to that of neurogranin, the most evaluated synapse-derived CSF biomarker for AD.^38^ Its longitudinal evaluation, especially in the late-presymptomatic stage, might be more valuable than cross-sectional measurements. It is promising that Ma *et al* 39 observed a correlation between NPTX1 in plasma and brain tissue; further studies are warranted to investigate whether NPTX2 can also be reliably measured in the blood, which would offer opportunities for longitudinal studies with larger numbers of samples.

We found an inverse correlation between NPTX2 and NfL in mutation carriers. NfL is a sensitive marker of neuroaxonal degeneration which is elevated in CSF and blood in the symptomatic stage of genetic FTD^40^ and in various other neurological disorders.^21^ Although a trend was found for symptomatic carriers alone after exclusion of ALS patients (who are known to have very high NfL levels),^21^ the lack of a stronger correlation probably reflects that NPTX2 and NfL are markers of different pathological processes which do not occur simultaneously.

Strengths of this study include the large sample size, despite the relative rarity of the disease, and the availability of corresponding clinical and brain imaging datasets. The inclusion of subjects with specific genetic defects allowed us to define pathologically homogeneous groups. Our NPTX2 findings are supported by similar results in NPTX1 and NPTXR, which correlated strongly with NPTX2, and indicate an overall reduction in NPTXs in genetic FTD.

The findings of this study must be viewed in light of some limitations. First, our longitudinal NPTX2 measurements were too limited in number to draw strong conclusions and require replication and more extensive statistical analyses in larger datasets. Second, using diagnostic criteria to label mutation carriers as presymptomatic or symptomatic may have failed to recognise subjects in a very early symptomatic stage. We calculated disease duration based on estimated time of symptom onset, rather than diagnosis, to ensure that any diagnostic delay did not affect correlative analyses. Third, three *C9orf72* mutation carriers had ALS without FTD, which, although increasingly recognised as part of the FTD spectrum,^1^ represents a clinically distinct phenotype. We ensured that these subjects did not affect our main results by repeating group comparisons after exclusion of these subjects. Finally, although brief medical and neurological history and examination did not reveal any significant neurological comorbidities, asymptomatic diseases, including cerebrovascular disease, could have confounded NPTX measurements. Future research focusing on potential confounding factors will be an important next step.

In conclusion, we provide evidence for NPTX2 as a novel CSF biomarker in genetic FTD. Its synaptic localisation and correlation with disease progression indicates that NPTX2 decreases probably reflect synaptic dysfunction or loss, providing novel opportunities for in vivo monitoring of synaptic integrity in genetic FTD. Treatment strategies aimed at improving synaptic connectivity may benefit from the use of NPTX2 as a tool to select and monitor patients with neural circuit dysfunction. More longitudinal data on NPTXs in presymptomatic and symptomatic mutation carriers might verify their value as (pre-)clinical biomarkers.
