# Supplementary material for: Neuronal pentraxin 2: a synapse-derived CSF biomarker in genetic frontotemporal dementia
Source: J Neurol Neurosurg Psychiatry. 2020 Apr 9;91(6):612–21. doi: 10.1136/jnnp-2019-322493 (PMC7279197; doi:10.1136/jnnp-2019-322493)
Supplement: Supplementary data [file jnnp-2019-322493supp001.pdf]

SUPPLEMENTARY FILE 1: SUPPLEMENTARY METHODS

NPTX2 concentrations were measured using an in-house ELISA. Briefly, 0.5 µg of rabbit anti-NPTX2 antibody in 50 mM Na_2CO_3 buffer (pH 9.5) was coated to a 96-well microtiter plate (Nunc) at 4°C overnight. The next day, after plates were blocked with 5% BSA at room temperature (RT) for 1 hour, 100 µl of the serially diluted NPTX2 standard proteins or CSF samples were added into wells and incubated at 4°C overnight with constant shaking. After plates were washed with TBS-Tween, 100 µl of biotinylated mouse anti-NPTX2 antibody was added and incubated at RT for 1 hour. After washing with TBS-Tween, 100 µl of HRP-conjugated streptavidin (Biolegend) was added and incubated for 1 hour. After washing with TBS-Tween, 100 µl of DAB substrate (Biolegend) was applied and incubated for 30 minutes at RT in the dark. In the end, 100 µl of 4 M H_2SO_4 stopping solution was added and the absorbance was measured at 450 nm. The absolute levels of NPTX2 in CSF were calculated from a standard curve.

NPTX1 and NPTXR concentrations were measured by Western blot. Briefly, human CSF was mixed with SDS loading buffer (2x: 125 mM Tris-HCl (pH 6.8), 4% SDS, 20% glycerol, 10% β-mercaptoethanol, 0.005% bromophenol blue) and heated at 70°C for 10 minutes. 10 µl of CSF were separated by 4-12% SDS-PAGE and transferred to PVDF membranes. After blocking with 5% non-fat milk, membranes were probed with primary antibodies overnight at 4°C. After washes with TBST (TBS with 0.1% Tween-20), membranes were incubated with HRP-conjugated secondary antibodies for 1 hour at RT. Immunoreactive bands were visualized by the enhanced chemiluminescent substrate (ECL, Pierce) on X-ray film and quantified using the image software TINA (www.tina-vision.net). Proteins migrating similarly in SDS-PAGE gel were assayed on different blots without stripping.

SUPPLEMENTARY FIGURE LEGENDS

Supplementary figure 1. NPTX2 plotted against age in the entire group of subjects (n=230). For blinding purposes, a jitter of ±2 years was applied to all subjects (analyses were done on raw data). The correlation coefficient was obtained from Spearman's rank correlation.

Supplementary figure 2. Receiver operating characteristic (ROC) curve to discriminate symptomatic from presymptomatic mutation carriers using NPTX2 (area under the curve=0.71; 95% CI 0.63-0.80).

Supplementary figure 3. Grey matter volume of the **(A)** frontal lobe, and **(B)** insula in presymptomatic (n=91) and symptomatic mutation carriers (n=35) and non-carriers (n=64), expressed as a percentage of total intracranial volume (TIV). Whiskers indicate minimum and maximum values. P-values are from Kruskall-Wallis tests with Bonferroni correction for multiple comparisons. ***p<0.001.

Supplementary figure 4. NfL concentrations in presymptomatic (n=106) and symptomatic mutation carriers (n=50) and non-carriers (n=70). Reported p-values are from ANCOVA with age as a covariate. Error bars represent median ± interquartile range. Orange squares indicate subjects who converted to the symptomatic stage during follow-up (n=3). Grey asterisks indicate subjects with amyotrophic lateral sclerosis (ALS) without FTD (n=3); grey crosses indicate subjects with both FTD and ALS (n=4). PRE: presymptomatic; SYM: symptomatic. *p<0.05; **p<0.01; ***p<0.001.

Supplementary figure 5. Relationship between changes in NPTX2 in longitudinal CSF samples (n=13) and time interval between CSF collections (r_s=0.116, p=0.705). Change in NPTX2 was defined as the difference in NPTX2 concentration between the first and the last CSF sample. Presymptomatic mutation carriers are shown as green squares; symptomatic mutation carriers as red triangles and converters are shown in orange. The non-carrier is shown as a blue circle. *GRN* mutation carriers are shown as open symbols, *C9orf72* mutation carriers as filled symbols and the *MAPT* mutation carrier as a half-filled symbol.

Supplementary figure 6. Relationship between NPTX2 concentration and subsequent annual change in phonemic verbal fluency among mutation carriers (n=118). Annualized change in phonemic verbal fluency was calculated by subtracting the score at CSF collection from the most recent score (collected through GENFI follow-up) and dividing by the time interval. *b* and p values are from linear regression analyses which included age, gender, center and education level as covariates.

Supplementary figure 7. Correlation between NPTX2 and **(A)** NPTX1, and (**B**) NPTXR in the entire group (n=230). Correlation coefficients are from Spearman's rank correlations.

Supplementary table 1. Relationship between NPTX2 and grey matter volume among non-carriers (n=64). Results were obtained through multiple linear regression with square-root transformed NPTX2 as the dependent variable, adjusting for age, gender and study site. Section A shows results when brain volumes were corrected for total intracranial volume; section B shows results using raw (uncorrected) brain volumes. Displayed p-values are before multiple testing correction; none were significant after Bonferroni correction. *b* indicates regression coefficient; β indicates standardized regression coefficient; SE: standard error.

A. Brain volumes as percentage of total intracranial volume			
	***b* (SE)**	**β**	**p**
**Whole brain volume**	-0.18 (0.42)	0.301	0.660
**Frontal lobe**	-1.18 (1.80)	0.187	0.515
**Temporal lobe**	1.88 (2.66)	0.422	0.484
**Parietal lobe**	1.27 (3.29)	0.314	0.700
**Occipital lobe**	0.62 (2.61)	0.205	0.814
**Cingulate gyrus**	1.65 (7.26)	0.215	0.821
**Insula**	-4.00 (19.89)	0.120	0.841
B. Raw brain volumes			
	***b* (SE)**	**β**	**p**
**Whole brain volume**	2.08E-5 (7.42E-6)	0.293	0.106
**Frontal lobe**	8.17E-5 (4.51E-5)	0.195	0.228
**Temporal lobe**	2.47E-4 (7.28E-5)	0.400	0.029
**Parietal lobe**	2.54E-4 (1.02E-4)	0.322	0.065
**Occipital lobe**	1.88E-4 (1.08E-4)	0.211	0.157
**Cingulate gyrus**	4.45E-4 (1.15E-5	0.200	0.184
**Insula**	1.02E-3 (9.16E-4)	0.169	0.271

Supplementary table 2. Relationship between NPTX2 and annualized changes in MMSE, TMT-B, phonemic verbal fluency, FTD plus CDR modules and CBI-R in mutation carriers. Annualized change was calculated by subtracting the score at the time of CSF collection from the most recently collected score and dividing by the time interval. Results are from linear regression analysis which included age, gender, study site and for cognitive tests education level as covariates. *b* indicates unstandardized regression coefficient; β indicates standardized regression coefficient; SE: standard error; MMSE: Mini Mental State Examination; TMT-B: Trail Making Test part B; CDR: Clinical Dementia Rating scale; CBI-R: Revised Cambridge Behavioural Inventory.

	**n**	***b* (SE)**	**β**	**p**
**MMSE**	136	0.001 (0.001)	0.159	0.077
**TMT-B**	107	-0.001 (0.004)	-0.040	0.693
**Phonemic verbal fluency**	118	0.004 (0.001)	0.302	0.001
**FTD plus CDR modules**	105	-0.001 (0.001)	-0.244	0.025
**CBI-R**	93	-0.004 (0.003)	-0.163	0.200
