# Supplementary figures and images for: Neuronal pentraxin 2: a synapse-derived CSF biomarker in genetic frontotemporal dementia
Source: J Neurol Neurosurg Psychiatry. 2020 Apr 9;91(6):612–21. doi: 10.1136/jnnp-2019-322493 (PMC7279197; doi:10.1136/jnnp-2019-322493)

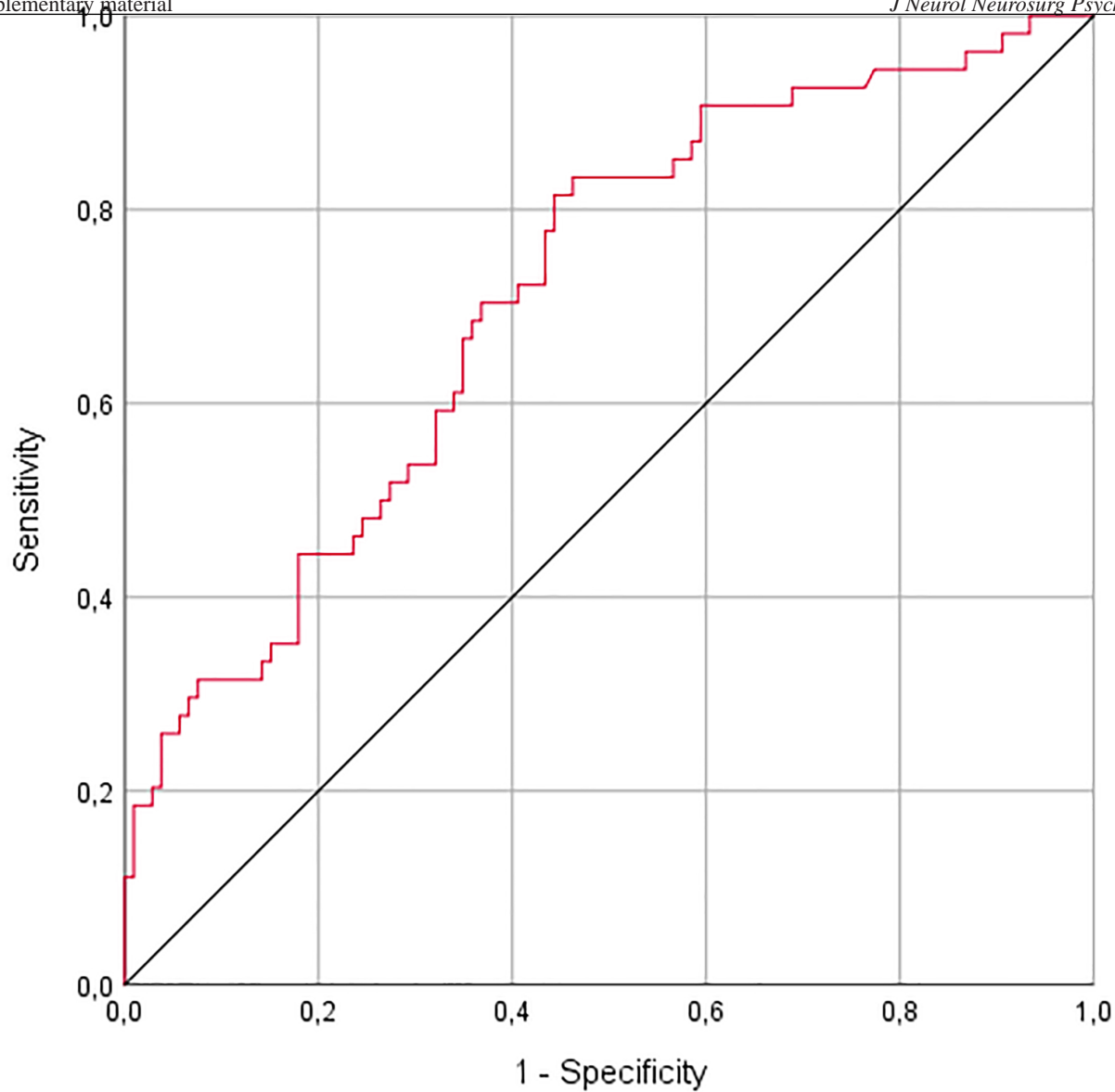

Supplement: Supplementary data [file jnnp-2019-322493supp003.pdf]

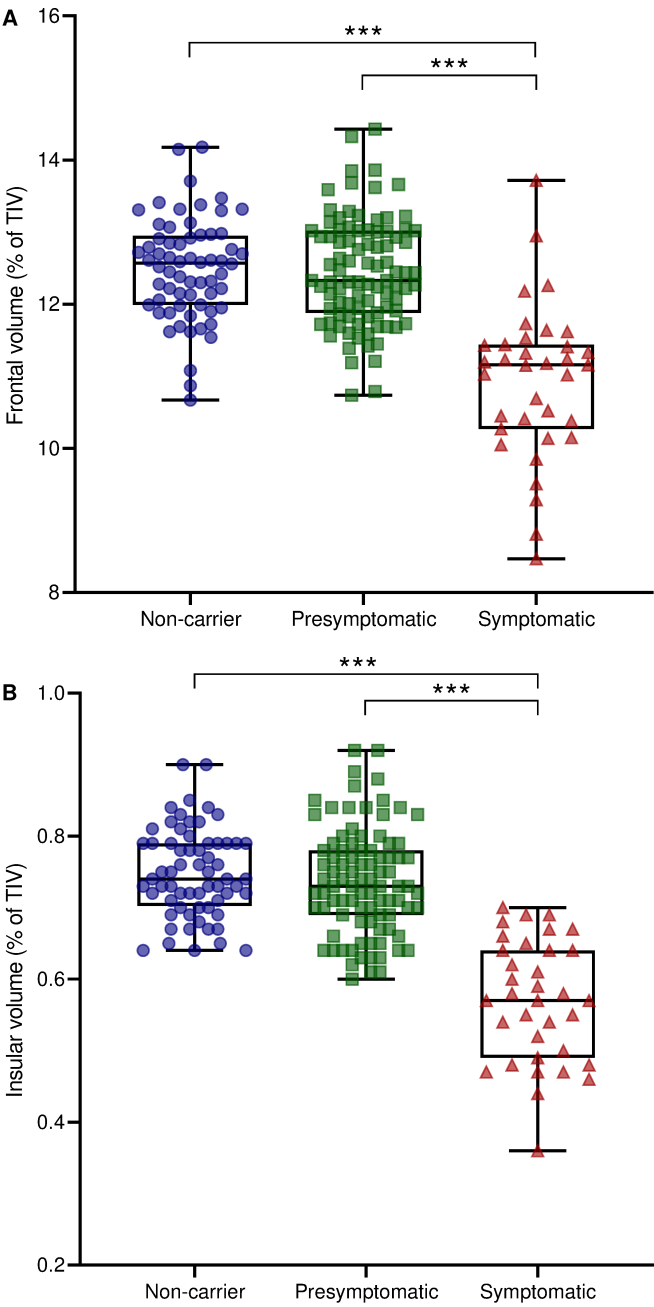

Supplement: Supplementary data [file jnnp-2019-322493supp004.pdf]

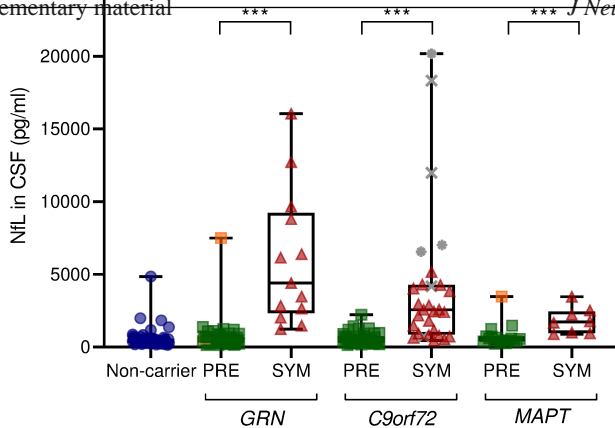

Supplement: Supplementary data [file jnnp-2019-322493supp005.pdf]

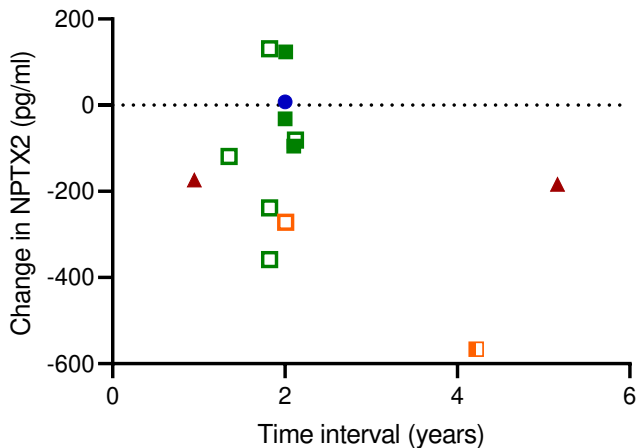

Supplement: Supplementary data [file jnnp-2019-322493supp006.pdf]

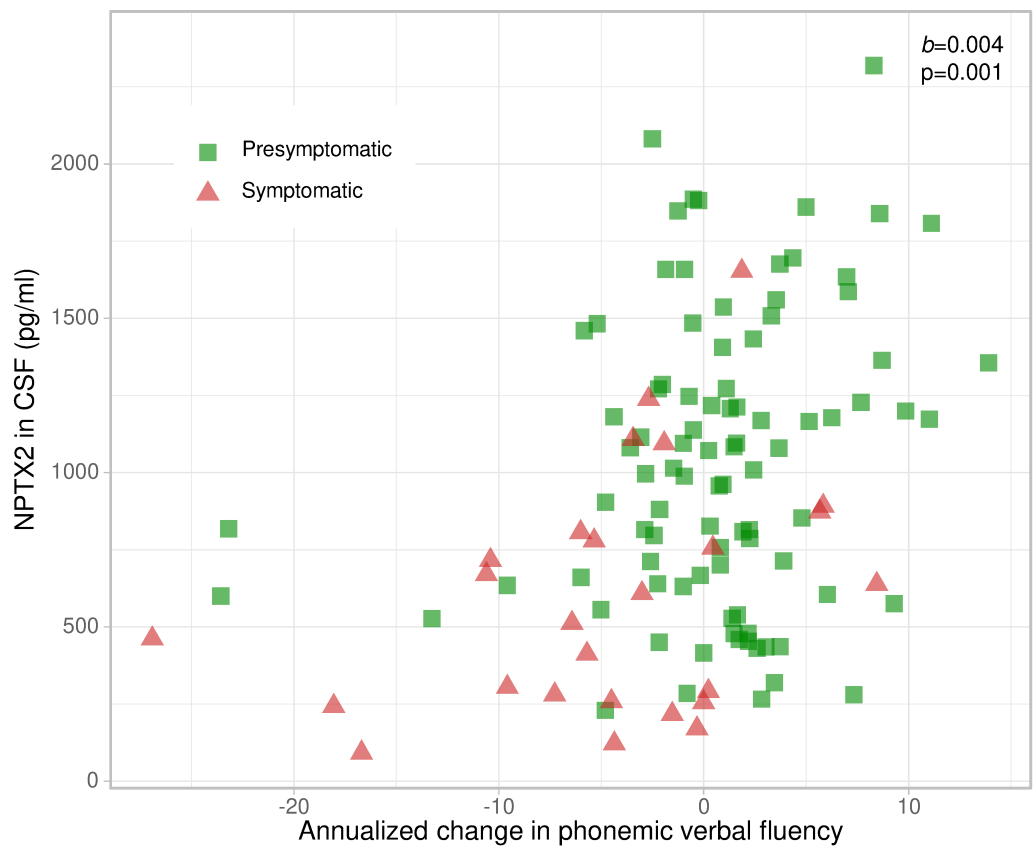

Supplement: Supplementary data [file jnnp-2019-322493supp007.pdf]

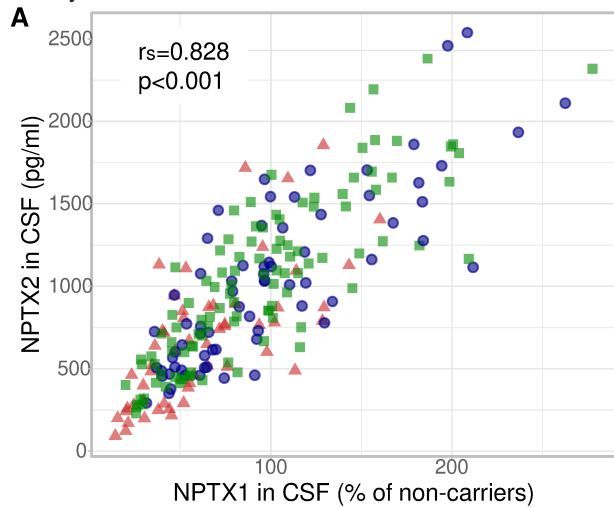

- Non-carrier
- Presymptomatic
- ▲ Symptomatic

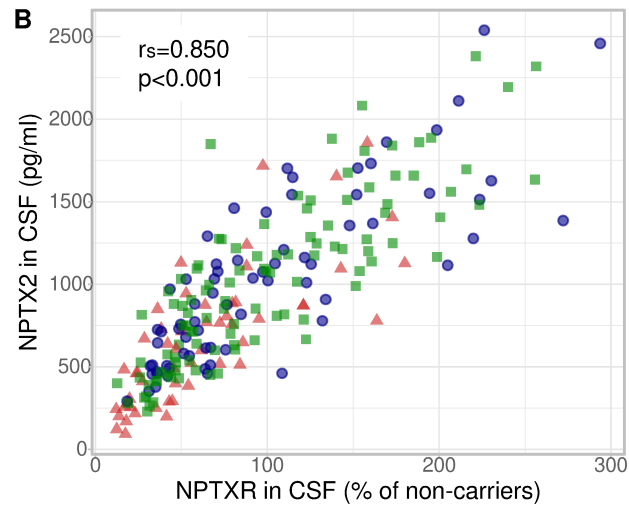

Supplement: Supplementary data [file jnnp-2019-322493supp008.pdf]
